# Physiological, aerodynamic and geometric constraints of flapping account for bird gaits, and bounding and flap-gliding flight strategies

**DOI:** 10.1016/j.jtbi.2016.07.003

**Published:** 2016-11-07

**Authors:** James Richard Usherwood

**Affiliations:** Structure and Motion Lab., The Royal Veterinary College, North Mymms, Hatfield, Herts AL9 7TA, United Kingdom

**Keywords:** Activation, Cost, Muscle, Economy, Efficiency, Wings, Work, Power

## Abstract

Aerodynamically economical flight is steady and level. The high-amplitude flapping and bounding flight style of many small birds departs considerably from any aerodynamic or purely mechanical optimum. Further, many large birds adopt a flap-glide flight style in cruising flight which is not consistent with purely aerodynamic economy. Here, an account is made for such strategies by noting a well-described, general, physiological cost parameter of muscle: the cost of activation. Small birds, with brief downstrokes, experience disproportionately high costs due to muscle activation for power during contraction as opposed to work. Bounding flight may be an adaptation to modulate mean aerodynamic force production in response to (1) physiological pressure to extend the duration of downstroke to reduce power demands during contraction; (2) the prevention of a low-speed downstroke due to the geometric constraints of producing thrust; (3) an aerodynamic cost to flapping with very low lift coefficients. In contrast, flap-gliding birds, which tend to be larger, adopt a strategy that reduces the physiological cost of work due both to activation and contraction efficiency. Flap-gliding allows, despite constraints to modulation of aerodynamic force lever-arm, (1) adoption of moderately large wing-stroke amplitudes to achieve suitable muscle strains, thereby reducing the activation costs for work; (2) reasonably quick downstrokes, enabling muscle contraction at efficient velocities, while being (3) prevented from very slow weight-supporting upstrokes due to the cost of performing ‘negative’ muscle work.

## Highlights

• The flight styles of many birds are not aerodynamically efficient.
• The implications of a simple physiological cost – muscle activation – are considered.
• Small-bird flight strategies reduce costs due to downstroke *power.*
• Flap-gliding reduces costs of downstroke *work* given constant ‘gearing’.
• Flap-gliding avoids ‘negative work’ during upstroke.

## 1 Background

### 1.1 General background

**Fig. 1:**
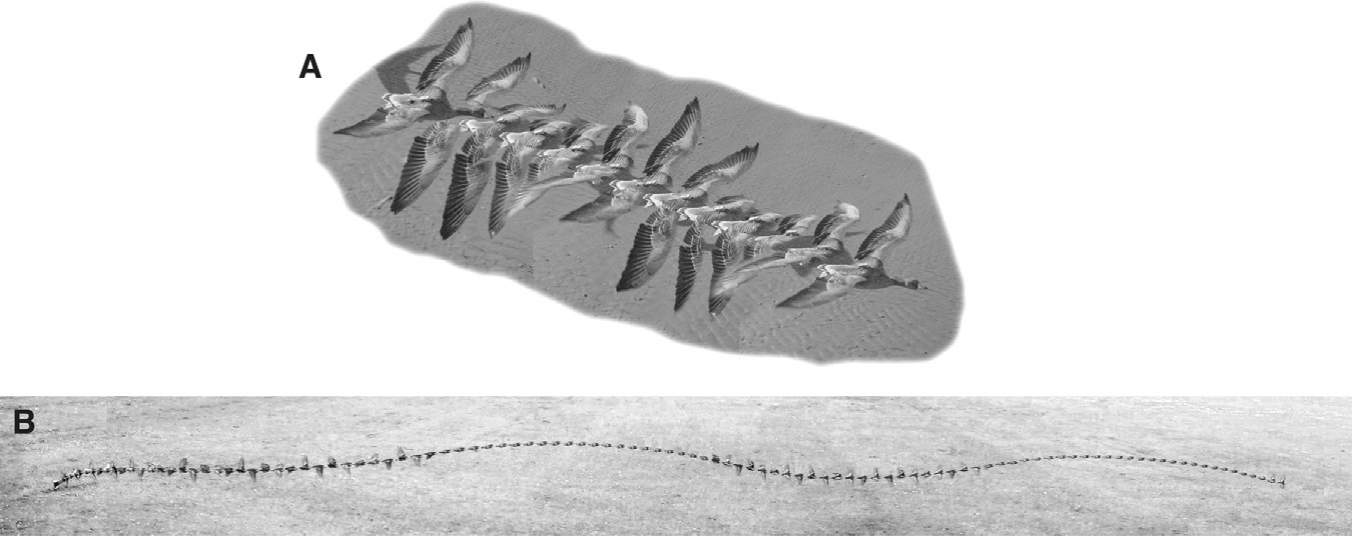
A qualitative demonstration of the scaling of flapping flight strategies. Larger birds fly more like aerodynamically efficient fixed-wing aircraft: the cruising greylag goose (A) supports body weight throughout the flap cycle with the outstretched wings flapping at low amplitude. In contrast, smaller birds (B, here a pied wagtail) deviate considerably from aerodynamically efficient gaits, with the wings providing minimal weight support for much of the time (both through an aerodynamically inactive upstroke and periods of near-ballistic, wing-folded ‘bounding’), and very high wingstroke amplitudes.

Flying birds show a range of different flight styles, even when travelling generally steadily. Domestic racing pigeons, geese, swans – medium to large birds – travel with continuous flapping (Fig. 1), with approximately constant frequency, amplitude and flight speed. In contrast, many bird species adopt some form of intermittent flight strategy. One form is commonly termed ‘bounding’, with periods of rapid, high-amplitude flapping interspersed with bounds, during which the wings are folded tightly against the body. Themes of the relationship between adoption of bounding and size, speed and morphology, are reviewed by Tobalske, 2001, Tobalske, 2010. Briefly, bounding is used only by small birds, with little owls and most woodpecker species (up to about 300 g) representing the larger end of the scale, down to a few grams in some parrots (budgerigars), wagtails, kinglets, finches and many other passerines. However, bounding is certainly not an absolute requirement for flying at small sizes: most waders rarely or never bound.

At medium and large sizes, an alternative intermittent flight style may be observed, with the body during periods between flapping being supported with outstretched, gliding wings. This style is termed here ‘flap-glide’, and is characteristic of many birds of prey even in generally direct, level flight. While flap-gliding is used by some large flying birds, many other large species – for instance geese and swans – flap continuously unless coming in to land.

Starlings highlight the challenge of making definitive rules concerning the distribution of bounding and flap-gliding: both intermittent flight styles are used (Tobalske, 1995), with only a quantitative shift from flap-gliding to bounding with increasing speed. Generally, however, bounding is limited to small birds, and for those small birds that do use it, bounds are more notable at higher speeds (Tobalske, 1995). Flap-gliding is more usually associated with larger birds, especially those competent at making use of thermals. However, flap-gliding is less related to size, with starlings, swallows and hawking dragonflies also frequently interspersing periods of flapping with glides, even in generally level flight.

### 1.2 Paper overview

In this paper, the implications of a physiological cost of muscle activation is considered, along with a range of aerodynamic and geometric constraints, to account for the potential energetic advantages and distribution of high-amplitude flapping and aerodynamically near-inactive upstroke (e.g. Spedding et al., 2003) and bounding of small, and flap-gliding flight in some larger, birds.

The paper begins (2) by making the case that current aerodynamic accounts for intermittent flight styles are unsatisfactory. Most aspects of this development are not novel, but do make the case more forcefully than previous analyses that neither bounding nor flap-gliding strategies offer a mechanical energetic advantage for birds that have evolved, or can adopt, appropriately sized wings. It continues (3) by considering the potential influence of muscle, and concludes that there is little support for a simple ‘fixed gear’ constraint. Instead, a ‘cost of muscle activation’ is proposed as a fundamental, general and revealing additional consideration that, along with various aerodynamic and geometric constraints, broadly account for the benefits and distribution of bounding (4) and flap-gliding (5) flight strategies. Further implications and predictions are then (6) explored.

## 2 Intermittent flight strategies deviate from work-minimizing ideals

To many people, it may be intuitive that efficient flight under ‘design’ conditions should be steady and level; after all, that is the flight style we are familiar with in any cruising passenger airliner. Further, in aircraft specialized for economy (a low fuel cost for a given load and distance) without any compromises for passenger comfort, steady, level flight is also used under cruising conditions. The case is re-made here not for novelty, but to emphasize an unresolved puzzle of bird flight: many birds adopt intermittent flight strategies inconsistent with aerodynamic or mechanical economy. To do this, fliers are treated initially as aircraft, and their aerodynamic power demands separated into their traditional components, broadly following Pennycuick (1989).

### 2.1 Parasite power

Parasite power is that required to overcome the drag of the body. All things (area, density, parasite drag coefficient) being equal, it is proportional to the cube of velocity *U*. It is then a simple mathematical result that any fluctuation of velocity *U* from the mean velocity $\bar{U}$ would result in higher mean power demands than steady flight, as

(1) $${\bar{U}}^{3}\leq \overset{¯}{U^{3}}.$$

### 2.2 Wing profile power

Profile power is conventionally that attributed to the drag of the wings; in flapping animal flight (where a component of profile drag may act to support body weight) profile power cannot always be elegantly divorced from induced power (below); but we shall continue here with the aircraft-style approach. Early attempts to find aerodynamic accounts for bounding flight rely on the benefit of reducing – eliminating – profile drag during periods of bounding, when the wings produce no or negligible lift. However, these approaches assume there are no additional profile drag cost during the flapping phases; this assumption, necessary if aerodynamic benefits of bounding are to be predicted, cannot be justified in detail. Clearly, if aerodynamic forces are to be produced by the wings over a small proportion of the cycle, the lift and thrust during flapping must be higher; and this can only be achieved with wings moving faster or with higher lift coefficients. If flapped faster, then similar issues apply as to parasite drag. If a bounding bird flaps supporting the body weight and providing the thrust for a proportion *q* of the time (sometimes termed the ‘power fraction’, Lighthill, 1977; Pennycuick, 2001), the aerodynamic forces (predominantly opposing weight) during this period would be 1/*q* that required for steady flapping. For a constant lift coefficient, the wing would have to move through the air more quickly to achieve this (as lift is proportional to the square of velocity), at $\sqrt{1/q}$ the speed. If the lift and profile drag coefficients were constant, then the power during flapping – proportional to the cube of velocity – would be proportional to ${\left(\sqrt{1/q}\right)}^{3}$, and the mean profile power through a full flap-bound cycle would be $\sqrt{1/q}$ that of steady flapping. For q≈1/2, appropriate for the pied wagtail of Fig. 1, this would indicate a 40% increase in mean profile power demand due to bounding.

If, instead, higher lift coefficients are assumed – allowing minimal kinematic differences during flapping whether bounding or not (e.g. Rayner, 1985) – then so are higher profile drag coefficients; profile drag coefficients cannot be considered independently from lift coefficients (see Withers (1981) and Spedding and McArthur (2010)). If a case is made that bounding is advantageous because it reduces profile power, it is, in effect, asserting that the wings are too large, and operating at an inappropriately low angle of attack and poor lift:drag ratio; and that something prevents the wings from being made conveniently smaller. Might bird wings be excessively large due to their function in slow or take-off flight? Might there be a mechanical limit or other aerodynamic cost to the extent of area reduction at higher speeds? Then bounding – adding periods where the wings contribute minimal drag – would allow the wings to operate more efficiently when they are producing aerodynamic forces. While such a concept may be coherent for flight at high speeds (Furber, in Lighthill, 1977; Rayner, 1977; Alexander, 1982; Ward-Smith, 1984; Rayner, 1985; Sachs, 2013) – that is, higher than appropriate for a given wing design – there has also to be the additional assumption that wing size cannot be conveniently controlled with partial retraction, as is often clearly adopted by birds in fast flight or flying into a strong headwind. Further, these models provide no account for the occurrence of bounding in slow, hovering or near-vertically ascending flight – or why bounding birds operate excessively large wings for their ‘normal’ flight.

### 2.3 Induced power

Induced power is that associated with accelerating a momentum jet of air downwards in order to provide weight support. In level flight it relates to the weight being supported *W*, the wing span *b* and velocity *U*:

(2) $$P_{\mathrm{ind}}\propto k_{\mathrm{ind}}\frac{W^{2}}{b^{2}\;U}$$

where *k*_ind_ is a factor greater that one to account for deviation from a steady, even, ideal momentum jet. Larger-amplitude flapping applies greater deviation from the ideal, steady momentum jet as the span fluctuates from full-spread, and air is accelerated in directions other than downwards (or, equivalently, aerodynamic lift vectors become inclined laterally – see Sachs (2015)). Fluctuations in – and near-complete loss of – weight support during upstroke and periods of bounding adds further deviation from the aerodynamic ideal. As an extreme case, with well-separated periods of weight support as in bounding, the effects of supporting weight for a proportion of the time *q* is clear. The weight supported during periods of flapping must compensate for the bounding periods – multiplied by 1/*q* – all be-it for a smaller proportion of the full flap-bound cycle (*q*). Thus, in this extreme case, the multiplying factor due specifically to bounding becomes (as also derived in Lighthill (1977), who acknowledges Mr. S. B. Furber):

(3) $$k_{\mathrm{ind}}={\left(\frac{1}{q}\right)}^{2}q=\frac{1}{q};$$

the pied wagtail of Fig. 1B, with a bounding duty cycle of approximately 0.5, thereby doubles the induced power demand from ideal. While this analysis is deliberately extreme – in truth, there may be some degree of weight support even during the bounding phase (Tobalske et al., 2009), and merging of closely spaced induced flows make this quasi-steady analysis an upper bound – any deviation from steady downwash generation directed away from the net aerodynamic force imposes higher-than-minimal induced power demands.

### 2.4 Inertial power

Flapping, reciprocating wings, unlike steadily revolving rotors, require cyclic changes in kinetic energy. The generation of the kinetic energy required to accelerate the wings may impose an energetic demand on the muscles. A rigorous inclusion of inertial power in an overall power calculation for flapping flight is challenging: is there any role of elasticity from muscle, tendon or feathers? Can the kinetic energy put into accelerating the wing (Alexander, 1977) be converted usefully into aerodynamic work as it slows (Ellington, 1984)? Might there be a muscle cost to slowing the wings (Van den Berg and Rayner, 1995)? Does a flexed-wing upstroke posture reduce rotational wing inertia sufficiently for energetic benefits? Despite these issues, it is possible to imagine that the conflicting requirements of inertial and aerodynamic power minimization might account for aerodynamically uneconomical high-amplitude, low-frequency flapping (Usherwood, 2009, Usherwood et al., 2011). However, an inclusion of inertial power does not provide an immediate account for intermittent flapping. To achieve a net force balance, periods of bounding with low aerodynamic force – assuming similar lift coefficients – must be compensated for with faster wing motions; faster flapping requires a greater inertial power, though only for the duration of flapping. The mean inertial power depends on the kinetic energy given to the wings each flap, proportional to the square of the maximum wing speed with respect to the bird centre of mass *V*_flap,max_, and the rate this work is performed *f*:

(4) Pinertia¯∝qVflap,maxf2

If this greater flapping velocity is achieved with a greater frequency – as appears to be the case from observations of scaling (Pennycuick, 2001), and is presumably demanded if flap amplitudes are already high – it would appear that intermittent periods of weight support would, if anything, also increase inertial power demands. Consider idealized hovering with a constrained wing stroke amplitude: flapping for half the time – *q*=0.5 – would require the wings, when flapping, (assuming constant aerodynamic force coefficients) to flap $\sqrt{1/q}$ or $\sqrt{2}$ times faster to produce the same weight support. To achieve this would require flapping the wings at a higher frequency – 1/*q* or 2 times higher. In the simplified hovering case, then, mean inertial power is proportional to 1/*q*; flapping only half the time doubles this cost. However, it should be noted that this is an incomplete analysis. The complexity of modelling the interplay between aerodynamic and inertial forces in flapping flight while including intermittent flight styles is beyond the scope of this paper; as is a thorough consideration of more complex aerodynamic models including interaction terms between the various sources of drag. These issues deserve further consideration, potentially using such computational approaches such as those developed by Parslew (2014).

## 3 Muscle constraints

The general conclusion appears to be (Ward-Smith, 1984, Rayner, 1985) that there is no simple, purely aerodynamic or mechanical account for intermittent flight strategies with sufficient generality to provide a satisfactory account for bounding in nature. Indeed ‘[aerodynamic] model predictions cannot explain the observation that some small passerine birds “bound” while hovering or during steep climbs. No aerodynamic factor can be responsible for this phenomenon’ (Rayner et al., 2001). Further, predictions of a purely aerodynamic benefit or neutral effect from flap-gliding (Ward-Smith, 1984, Rayner, 1985) requires model assumptions that should now be questioned, especially with the aerodynamic principles laid out above. The finding that aerodynamic power considerations can account for flap-gliding with an ‘undulating’ up-down flight profile can be attributed to a predicted reduction in induced power with ascending flight (Rayner, 1985; page 64, Eq. (9)). However, this reduction in power appears excessive due to the claim that the ‘weight support required is reduced’ (during steady ascending flight) – an assertion that is not adopted in other analyses of ascending flight in animals (e.g. Wakeling and Ellington, 1997) and appears erroneous. Expanding this point, the calculation required can be described using the analogy of a person walking ‘up’ a ‘down’ escalator. When walking at a speed that maintains height, work is continuously put into the escalator by the person (meaning that – in our otherwise lossless idealized system – the escalator must apply brakes to dissipate this energy), analogous to the bird supplying energy to a downward momentum jet of air in order to support body weight in level flight. Now consider the demands of walking steadily but a little faster up the escalator which (for the moment) has not changed speed. In this case, the rate of work being applied to the escalator remains the same (body weight multiplied by the vertical component of escalator belt speed), and some additional power is required for the person to perform work against gravity. To add a slight complication, if the speed of the escalator is a direct analogue of the induced downwash jet velocity *V*_ind_, then it actually slows somewhat due to the higher air velocity. However, the weight being supported is not reduced in steady climbing flight, so the induced power expression of Rayner considerably overestimates the induced power reduction in climbing, and there appears to be no purely aerodynamic case for undulating flight.

**Fig. 2:**
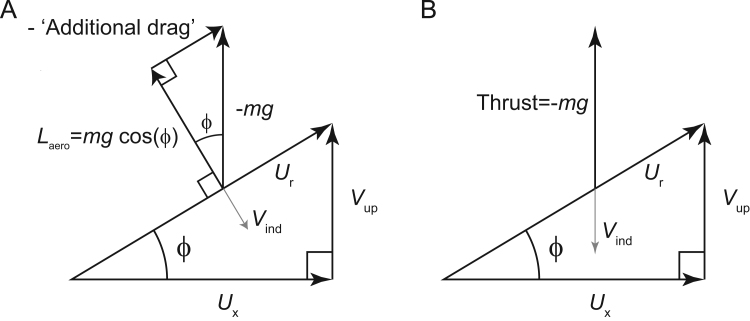
The aerodynamic geometries used for calculation of induced power in ascending flight at an angle ϕ, upward velocity *V*_up_, horizontal velocity *U*_x_ and resultant velocity *U*_r_. Undulating flight has been described as providing energetic cost reduction through reduction in induced power demands (Rayner, 1985), presumably due to the rotation of the aerodynamic lift vector *L*_aero_ – defined as acting perpendicular to the resultant air velocity *U*_r_ – and therefore its reduction by a multiple of cos(ϕ) (A). However, in Rayner's formulation, an additional aerodynamic force is required to result in net weight support (-*mg*), which is treated as an additional drag. Rayner assumed that small angle assumptions allow the power demands of this additional drag to be treated simply – as the additional drag force multiplied by the resultant velocity. Problematically, this asserts that the counteraction of the additional drag force (that, in part, supports body weight) can be achieved without any costs associated with accelerating air. An alternative approach to calculating induced power demands is to treat the net aerodynamic force required as a thrust – and not to treat aerodynamic lift in isolation (B). With this approach, weight is supported and the induced velocity *V*_ind_ required for the weight-supporting momentum flux is appropriately vertical; and any purely aerodynamic benefit to undulating flight is lost.

This does raise the question of why Rayner claimed flying at an incline reduces the weight support requirement. The answer may be as shown in Fig. 2: that a rotation of the resultant air velocity from horizontal to inclined by an angle ϕ would rotate the orientation of the aerodynamic lift vector – defined as being perpendicular to the relative air velocity – and so reduce its magnitude; and that Rayner's ‘weight support’ actually referred to aerodynamic lift force. If this rotation is used to justify the reduced induced power calculation, it assumes that the aerodynamic force required in addition to aerodynamic lift in order to achieve net weight support and zero horizontal acceleration (termed in Fig. 2A and treated in Rayner (1985) as ‘additional drag’) can be achieved without aerodynamic losses. However, if the vertical forces in climbing flight are achieved with broadly the same aerodynamic mechanisms as level flight, then presumably the same inefficiencies – due to producing a momentum flux by accelerating a finite mass of air with finite-length wings – applies approximately equally. Thus, it is the vertical force – or ‘Thrust’ in Fig. 2B and following Wakeling and Ellington (1997) – and not the aerodynamic lift force, that should be used in calculating induced power demands of constant velocity flight, even at an incline. It is therefore appropriate and timely to revisit consideration of whether simple physiological constraints or cost functions might provide some insight (Ward-Smith, 1984, Rayner, 1985).

### 3.1 ‘Fixed-gear’ and ‘maximal activation’ hypotheses

The predominant muscle constraint considered in terms of bounding strategies is that the muscles of small birds might, for some reason due perhaps to details of innervation or muscle geometry, be constrained to contracting near-maximally in some parameter, presumably related to power or efficiency. Thus, a bird able to flap rapidly and powerfully for take-off might adopt an intermittent, bounding flight when cruising, keeping each muscle contraction broadly similar and making use of the bounding duty cycle *q* in order to modulate mean aerodynamic power output (Rayner, 1985, Pennycuick, 2001). Such constraints have (confusingly) become termed both ‘fixed gear’ (e.g. Tobalske and Dial, 1994; Tobalske et al., 1999, 2005) or ‘continuous gear’ (Rayner et al., 2001). One prediction of this concept (Rayner, 1985) is (during flapping in small, bounding birds) a uniformity in wing kinematics over a range of speeds and the recruitment of all the pectoralis muscle during each downstroke. However, empirical observations do not support these predictions in zebra finches, budgerigars or starlings (Ellerby and Askew, 2007, Rayner et al., 2001, Tobalske and Dial, 1994, Tobalske et al., 1999, Tobalske et al., 2005). The question therefore remains as to what physiological feature, presumably of muscle, might combine with flapping aerodynamics to account for intermittent, bounding strategies; and why this should apply especially – but not universally – to small birds.

### 3.2 Cost of muscle activation for work and power

Muscle activation is physiologically costly, even when the muscle itself produces zero mechanical work or power. The energetic costs associated with activation – due in part to pumping calcium ions to restore the electrochemical gradient after a contraction (Barclay, 2012) – may be significant, apparently dominating the physiological demands of steady, level locomotion (Pontzer, 2007, Pontzer, 2016, broadly extending observations of Kram and Taylor (1990)). However, strategies that reduce muscle activation may conflict with those that minimize work, and have scaling implications. This concept has been applied recently to provide a qualitative account for the scaling of posture in terrestrial animals (Usherwood, 2013), and quantitative accounts for the scaling of walking and running mechanics in children and adults (Hubel and Usherwood, 2015). It should be highlighted that the ‘power’ cost concept used here is that during muscle contraction, and not the mean power of flight.

Here, the assumption is made that there is little scaling of muscle efficiency directly in terms of cross-bridge cycling, so this can be neglected despite the uncertain, but potentially relatively large, physiological cost associated with this component of muscle contraction. Instead, the focus is on the costs of muscle activation. A key assumption is that the fundamental demands for activation come from the work and power requirements over a contraction. This assumes that the system is ‘well-tuned’ – that is, it has an appropriate combination of mechanical advantages resulting in an overall Effective Mechanical Advantage between muscle in-lever *r* and mechanical out-lever *R* (EMA=*r*/*R*; Biewener, 1989). Costs associated with only muscle force (for instance during gliding) are assumed to be low or negligible (despite constraints to adjustments of *EMA* flap-glide – see Section 5): isometric muscle forces are assumed to be achievable with lower muscle activation demand (consistent with muscle force-velocity measurements) than shortening muscles; further, specialised muscle physiology may sometimes result in additional reduction in isometric force costs (e.g. Rosser et al., 1994). If muscle is indeed activated predominantly to meet work and power demands, the contrasting scaling of these parameters due to changes in contraction duration provide a general mechanistic account for scaling of posture (Usherwood, 2013) and an approximate quantitative account for the scaling of human gaits through ontogeny. It is proposed here that similar issues account for the scaling of flapping gaits and strategies, including the high wing-stroke amplitude, lack of weight support during upstroke, and periods of bounding characteristic of many (though not all) small birds.

If the physiological cost due to activation relates to activated muscle volume, and this volume is indeed fundamentally demanded from work and power requirements *during a contraction*, a key property of muscle can be expressed in terms of time. If muscle can be taken as having a finite work and power capability averaged over a contraction – for example 50 J/kg and 500 W/kg – then this imposes a time boundary (in this case, 0.1 s). Any contraction with a duration greater than this time requires activation of muscle primarily to provide work; the power demands will be met anyway. Conversely, any contraction with duration less than this time requires muscle activation primarily due to power; the work demands will be met anyway – no muscle volume has to be activated for work *per se* as such a large volume must be activated to produce the power. This insight goes some way in accounting for why many small animals, with contraction durations less than 0.1 s, adopt gait strategies that reduce power demands, even if this comes at the cost of increased work (Hubel and Usherwood, 2015). The argument as applied here does not require that muscle activation costs are the only ones contributing to metabolic cost; merely that these costs are of sufficient magnitude to be physiologically relevant (see Homsher et al., 1972; Smith, 1972; Barclay, 2012). To expand on this point, let us assume that the physiological activation cost *C*_work_ of muscle required to meet work demands *W*^+^ depends on the volume of muscle that has to be activated – dependent on the muscle mass specific work capability *E*^⁎^ and the physiological cost (energy demand) per mass of activated muscle *k*_act_:

(5) $$C_{\mathrm{work}}=k_{\mathrm{act}}\frac{W^{+}}{E^{*}}$$

Further, assume the cost *C*_power_ of activating due to power *P*^+^ during the contraction depends on the specific muscle power capacity (power/kg, or P^*^)

(6) $$C_{\mathrm{power}}=k_{\mathrm{act}}\frac{P^{+}}{P^{*}}.$$

The activation costs *C* are expressed here with dimensions of energy (Joules), the physiological cost associated with activating the required mass of muscle to meet a given mechanical demand.

Given the power during contraction relates simply to the work and duration of contraction *T*_act_, it can be seen that a muscle contracting over less than a duration of E*/P* must be doing so in order to provide the power demand:

(7) $$\frac{C_{\mathrm{power}}}{C_{\mathrm{work}}}>1$$

if

(8) $$\frac{P^{+}}{P^{*}}>\frac{W^{+}}{E^{*}},\;\mathrm{so}$$

(9) $$\begin{array}{c} W^{+}\\ {\begin{array}{c} P \end{array}}^{*}T_{act} \end{array}>\begin{array}{c} {\begin{array}{c} W \end{array}}^{+}\\ {\begin{array}{c} E \end{array}}^{*} \end{array};\;\mathrm{that}\;\text{is},\;\mathrm{when}$$

(10) $$T_{\mathrm{act}}<\frac{E^{*}}{P^{*}}\approx \frac{50J/\text{kg}}{500W/\text{kg}}=0.1s.$$

This would indicate that a muscle contracting for less than 0.1 seconds would have a higher volume activated than necessary to meet the demands of work alone. Note that the 0.1 s value relating to 500 W/kg, 50 J/kg, or a 30% strain contraction at 3 lengths/second, is merely empirically reasonable (see, for instance, Channon et al. (2012)), and its exact value is unimportant to the qualitative development presented here. Clearly, smaller species can have ‘faster’ muscles, but this comes at a cost of ‘proportionately greater expenditure of energy’ (Hill, 1950). Costs due to high power demands (due to brief periods for performing work during muscle contraction in downstroke) may therefore be reflected in either high volumes of activated muscle, or in maintaining and operating higher-velocity muscles. The argument is developed here assuming that muscle properties are broadly scale-invariant, but an equivalent case could be made given a cost to maintaining muscles of high strain rate: strain ratio. Other factors determining the duration of effective muscle contraction may play a role (e.g. deactivation rate; Marsh, 1990); again, however, mechanisms enabling faster muscles come at a physiological cost that might be ameliorated if kinematic strategies were possible to lessen the scaling of timing with size.

When applied to flapping flight, the same scaling issues apply as to terrestrial gaits if we can assume that muscle and external (aerodynamic) moment arms are suitable tuned. The premise of this paper is that smaller flapping birds forego many aspects of mechanical efficiency in order to increase the duration of muscle contraction, providing time to reduce the muscle activation demand of mechanical power (during the contraction). In steady, level flight, (where no net mechanical work is being performed on the centre of mass), the use of the term ‘efficiency’ may require some clarification. Aerodynamic efficiency is usually defined with the ‘useful work’ being that required to maintain a theoretical ideal wing of given span, speed and weight, divided by the ‘actual work’ required to overcome drag. In flapping flight, it would appear appropriate to add any additional inertial costs to the denominator term.

## 4 Applying ‘cost of activation’ to account for bounding

While there may be a moderate scaling of muscle properties with size (e.g. Seow and Ford, 1991; Tobalske, 1996) especially if insect muscles are also considered (see Section 6.1), such scaling is not extreme in vertebrate muscles: Seow and Ford (1991) report shortening velocities scaling with body mass^−1/8^ in mammalian muscles. Consider first an ibis flapping at a frequency of 5 Hz and downstroke duration of approximately 0.1 s. In this case, if the work:power capability of muscle was 0.1 s, any volume of muscle would be activated to provide equally both the work and power demands. Next consider a zebra finch flapping at 28 Hz (Tobalske et al., 2005) and a downstroke or pectoralis contraction duration of approximately 0.02 s. It is inconceivable that zebra finch muscle is five times faster, or capable of producing the same mass-specific work at a fifth of the strain, than an ibis muscle. This means that a greater volume of zebra finch muscle must be activated to meet the power demands during the contraction than is necessary for work. If muscle activation itself comes at a metabolic cost, one might expect strategies to limit this power demand.

### 4.1 Why cannot small birds use small amplitude flaps with a downstroke of 0.1 seconds?

**Fig. 3:**
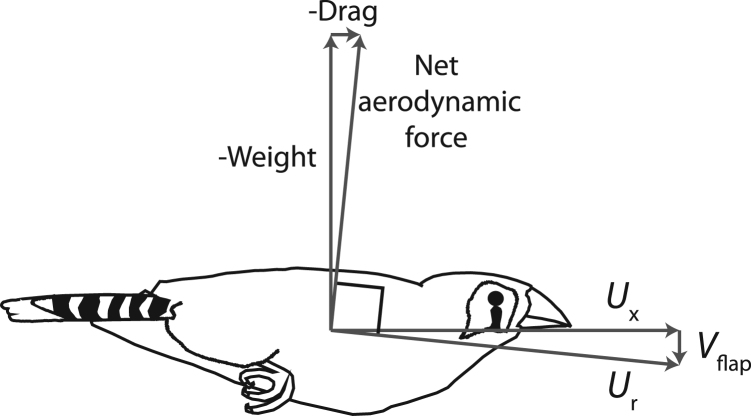
The geometric demands of producing the required net aerodynamic force to overcome body drag and oppose body weight using predominantly aerodynamic lift of the wings during downstroke only. Aerodynamic lift acts perpendicular to the wing velocity through the air *U*_r_; in order to achieve this orientation, the flapping velocity of the wing *V*_flap_ cannot be below approximately *U*_x_ drag/weight. The 1:10 geometry is appropriate for a zebra finch at approximately 14 m/s (Tobalske et al., 1999, from which the finch image is also taken).

Low-amplitude flapping would allow minimal deviation from aerodynamic work-minimizing flight; contraction over at least 0.1 s would prevent muscle activation for power demands undemanded by work. What prevents small birds from adopting this strategy? The issue is that the net direction of the aerodynamic forces produced by the wings must be orientated appropriately: the required net aerodynamic force is dominated by weight support, but parasite drag from the body also demands a net thrust from the wings, which proportionally increase at smaller sizes and higher speeds. This geometry imposes constraints on wing kinematics if force production is dominated by aerodynamic lift, as this force acts perpendicular to the relative airflow (Fig. 3). Flapping geometry is therefore constrained by the required aerodynamic force vector and flight speed if the wing produces lift predominantly with net low pressure only over the dorsal surface. A bird flapping downwards at too low a velocity will be unable to orientate the aerodynamic lift vector sufficiently forward. This imposes two issues – one physiological, the other aerodynamic/geometric – for small flapping birds: the desirability of relatively long-duration muscle activation (~0.1 s), yet the geometry that requires the wing to move quickly downwards to orientate aerodynamic lift to produce sufficient thrust. These issues combine to give a novel account for the aerodynamically uneconomical features of small-bird flapping flight. The geometric issue is shown in Fig. 3, and can be expressed mathematically for small angles.

For small angles (relatively high weight support v.s drag opposition), the minimum flap velocity *V*_flap_ allowing the geometry to produce the required net aerodynamic force at a forward flight speed *U*_x_ is given by:

(11) $$\left|V_{\mathrm{flap}}\right|\approx \left|U_{x}\right|\frac{\mathrm{Drag}}{\mathrm{Weight}}.$$

This geometric reduction is clearly wrong in detail – what with time-varying aerodynamic forces, finite stroke plane amplitudes and spread of chords along the span of the wing. However, the principle is sound: wings that produce aerodynamic forces predominantly on the downstroke are constrained in how slowly they may flap in order to produce sufficient thrust to oppose drag. And the minimum flap speed goes up with flight speed and parasite drag. Note that this geometry is, while similar, not identical to the issues of advance ratio in propeller theory (Mises, 1945; see Vogel (1994)). Real propellers with a finite lift:drag ratio cannot operate very slowly on an aircraft moving quickly: the resultant force fails to provide a net thrust. But consider a theoretical, ideal propeller able to produce only pure aerodynamic lift – perpendicular to the resultant airflow. Such a propeller would (theoretically) be capable of producing net thrust at near-infinite advance ratio – at very high flight speeds or low tip speeds. The same is not true in the flapping case: even with a theoretical, ideal wing capable of producing pure aerodynamic lift, expression (11) stands, and very high flight speeds or low flap speeds are precluded. In effect, the minimum propeller speed is limited by the lift:drag ratio of the propeller aerofoil, whereas the minimum flap speed is related to the weight:drag ratio of the body.

A high wingstroke amplitude (1) provides a greater duration for muscle contraction for a given wing velocity, reducing muscle activation demands due to power during the contraction; (2) ameliorates the geometric issues described above somewhat: away from horizontal, mid-downstroke, a smaller component of aerodynamic lift acts to support body weight, allowing some further forward-tipping of the average resultant aerodynamic force vector. Weight support with outstretched wings during upstroke, while potentially improving aerodynamic efficiency, is problematic in such high-amplitude flapping as this would also be associated with a (albeit small) degree of drag, tipping the net aerodynamic force vector backwards, making the task of achieving appropriate downstroke geometry even more challenging.

Let us also suppose that there is some minimum functional lift coefficient. While this may not be identical to that providing the best lift:drag ratio (because the argument here is not only concerning aerodynamic economy), very low lift coefficients would still be energetically unfavourable from an aerodynamic perspective. Let us now review the three key assertions:

1) a physiological cost to muscle activation to provide power during a brief contraction applies pressure towards relatively long downstroke duration in smaller birds
2) geometric constraints of providing net thrust requires high-speed downstrokes. Combined with (1), this results in a pressure for high amplitude downstrokes, especially at small sizes and high flight speeds
3) functional lift coefficients are constrained.

With these three points, we can now account for the ‘excess’ aerodynamic force produced during flapping phases of bounding birds. The required adjustment to achieve an appropriate net force balance is then clearly the net aerodynamic duty cycle – a combination of downstroke: upstroke duration and flap-bound duty cycle *q*. If wings during downstroke are driven to provide high aerodynamic forces from physiological (1), geometric (2) and aerodynamic (3) demands, extended periods of aerodynamically inactive upstroke – including periods of bounding – are required to permit the bird to maintain a steady average flight speed.

An alternative way of viewing the same concept is to answer why bounding birds flap with excessively long wings. Shorter wings would allow aerodynamically adequate lift coefficients (3) and reduce the aerodynamic impulse each flap, thereby reducing the demand for aerodynamically inefficient long bounding periods. However, to achieve the required flap velocity from the geometric constraints of producing thrust (2), the angular velocity of the shorter wings would have to be higher; given constraints to swept flap angle (approximately 180°), the downstroke and muscle contraction durations would then have to be briefer. ‘Excessively’ long wings – those resulting in excessive aerodynamic impulse each downstroke that has to be balance by periods of aerodynamically inactive upstroke or bounding – thereby extend the duration of muscle contraction and limit the wasteful muscle activation costs demanded from power during the contraction.

At larger sizes, where downstroke duration – and the dominant powering muscle contraction duration – approaches 0.1 s, the pressure to adopt aerodynamically uneconomical gaits or strategies in order to ‘buy’ time to perform work without excessive muscle power diminishes. A 0.1 s downstroke and approximately even down/upstroke timing would equate to a steady flapping frequency of 5 Hz. This happens to match the wingbeat frequency of the pileated woodpecker, in which periods of bounding are marginal (Tobalske, 1996).

### 4.2 Contrasts with the Fixed-gear hypothesis

Both the ‘Fixed-gear hypothesis’ and the current suggestion based on muscle activation costs identify flap-bounding as a means for manipulating the overall duty cycle of wing action. Both assume that this use of duty cycle for modulation is due to some constraint in downstroke velocity. However, the drivers and implications of the constraints contrast. The fixed-gear hypothesis suggests that wing velocity is driven by muscle velocity; that the range of effective muscle velocities is limited; and predicts the wing flap velocity should remain approximately constant across speeds. In contrast, the geometric constraint of Fig. 3 and expression 11 suggest that wing velocity is constrained by aerodynamic/geometric considerations. In this case, the wing velocity during downstroke is predicted to increase with both flight speed and parasite drag.

## 5 Applying ‘cost of activation’ and other constraints to flap-glide strategies

While the case has been made here that larger birds adopt a cruising flapping style close to that expected in terms of aerodynamic efficiency, some do adopt a form of intermittent flight that does not fit this view: flap-gliding. For instance, many raptors can be readily distinguished from birds of similar size due to their periods of gliding interspersed between phases of flapping, even during generally steady, direct flight. Can the simple cost to muscle activation provide insight into the potential advantage of this flight style, and its distribution among birds? Here, we propose a series of constraints imposed by muscle and geometry to account for deviation from the low-amplitude, constant weight-support aerodynamic ideal. In this development, unlike for bounding and terrestrial gaits, the ‘gearing’ or Effective Mechanical Advantage between muscle and external (aerodynamic) forces are assumed to *not* to be able to freely and appropriately tuned.

### 5.1 Muscle constraint 1: strain

**Fig. 4:**
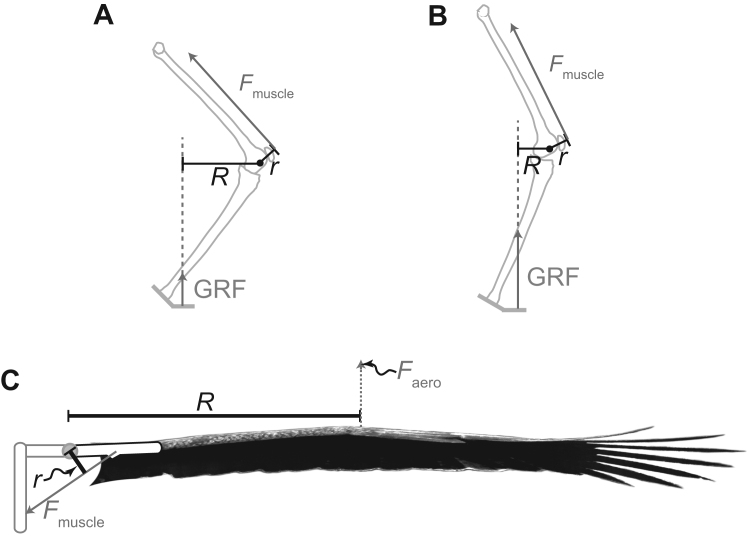
Schematic geometries of terrestrial (A, B, adapted from Biewener et al. (2004)) and flying (C) moment arms, and the contrasting potentials for the manipulation of Effective Mechanical Advantage (EMA) – the ratio of in-lever (the muscle force *F*_aero_ moment arm, *r*) to out-lever (the Ground Reaction Force (GRF) or Aerodynamic Force (*F*_aero_) moment arm, *R*). Large terrestrial animals are capable of extreme changes of EMA with subtle changes in posture because the GRF passes close to joint centres. Flying animals have less capacity for varying EMA because the centre of pressure – the origin of the vector *F*_aero_ – lies a long way from the joint centre. Further, changes in EMA through changes in centre of pressure location appears unfavourable: more rapid flapping would drive the outer wing faster, result in a relatively more distal centre of pressure and in effect increase the ‘gearing’ (reduce the EMA) just when the muscle should be acting at high power and velocity. Flapping the wings in cruising flight at a much lower amplitude than during high-power activities (take-off, load carrying etc.) would therefore allow only small muscle strains, and small work capacity per muscle per activation. A metabolic cost to activation might prohibit such low-strain contractions, albeit at some cost in terms of greater deviation from aerodynamic efficiency ideal.

In recent considerations of the implications to the costs of muscle activation, applied to terrestrial locomotion, it has been assumed that simple morphological or postural adjustments can change moment arm ratios so as to make ‘gearing’, ‘transmission ratio’ or ‘Effective Mechanical Advantage’ (following Biewener (1989)) a free parameter, and the muscle activation demands only related to the work and power requirements (Usherwood, 2013, Hubel and Usherwood, 2015). This is likely to be less true for flapping birds that fly over large range of flight speeds, loads etc. (Fig. 4): while the weight-support vector passes close to joint centres in large terrestrial animals, allowing the ‘out-lever’ to be shortened or lengthened considerably with only minor postural adjustments, the equivalent out-lever for birds is the distance between shoulder centre of rotation and, for a horizontal wing, the aerodynamic centre of pressure. Manipulation of moment arm ratios is therefore much more constrained in flapping flight. What is more, movement of the centre of pressure would tend to act unfavourably. Consider take-off: just when a ‘low gear’ or high EMA would be beneficial, the relatively high velocity of the wing tips tends to move the centre of pressure distally, creating a ‘high gear’, low EMA.

If there are indeed limits to the changes in mechanical advantage, then a muscle capable of providing the power and work of highly energetic periods of flight (including slow and take-off flight or load-carrying) would experience only very low strains during low-amplitude cruising flight where economy was desirable. Low muscle strains result in a lower work capacity E^*^ for a single contraction of a given mass of muscle; activation costs to provide work are thus higher (see Eq. (5)) if wing amplitudes – and so muscle strains – fall too low. While this may be ameliorated to a certain extent in those large birds that carry and operate muscles with different fibre types and *EMA* depending on context (e.g. Rosser et al., 1994) there is presumably also a cost – especially in flying animals – to maintaining and carrying multiple powering systems with different specialisations.

### 5.2 Muscle constraint 2: strain rate

Again, assuming that bird flight muscles are, at times, capable of high-power, high-work contractions, operating the muscles at very low strain rates is inefficient (note that this is a similar assumption as has been used previously in the ‘fixed-gear’ hypothesis of flap-bounding flight). Strain rates much below 0.2V_max_–0.4V_max_ result in a low thermodynamic efficiency (Reggiani et al., 1997, He et al., 2000). So, with the assumptions that bird muscles are capable of producing high work and power on occasion, and are unable to alter their mechanical advantages sufficiently, simple muscle costs provide some account for the finite-amplitude downstrokes over reasonably brief periods. Why, though, are upstrokes not slow?

### 5.3 Muscle constraint 3: a negative-positive- work cycle, while netting no change in energy, demands at least the positive work component to be provided by muscle

**Fig. 5:**
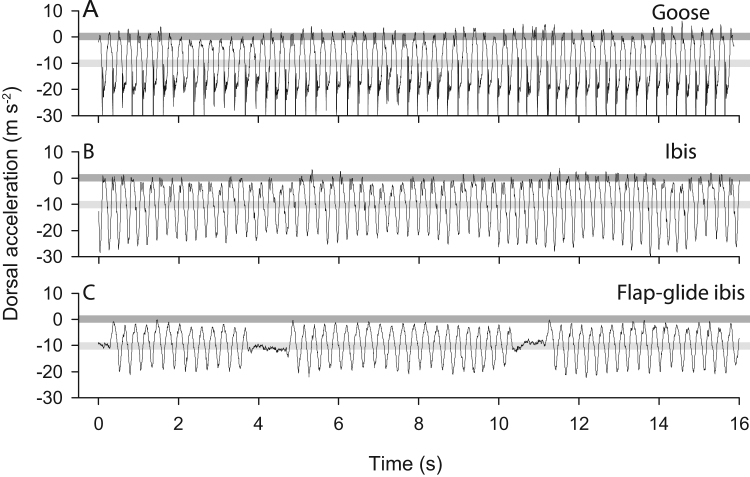
Example dorsal accelerations of back-mounted sensors during bird flight at approximately 15 m/s. A: greylag goose in a flock following a car; B: a Northern bald ibis in a flock following a para-wing; and C: the ibis towards the end of the flight showing flap-gliding. No filtering, adjustments or rotations are performed: the dorsal orientation is assumed to be vertical, and some of the high-frequency signal components may be attributed to the less-than-rigid mounting. A value of −9.81 m s^−2^ would be observed with a static sensor (light grey background line; in this case, it measures gravity); a value of zero indicates free-fall (darker grey background line). In both geese and ibis, the body experiences accelerations close to free-fall during the upstroke, indicating that the vertical aerodynamic forces on the wings act to accelerate the wings up, and this is not resisted (negative work is not performed) by the shoulder.

While slow upstrokes maintaining weight support over the amplitude determined by the above downstroke constraints may minimise net aerodynamic work, the demand on the muscles can be high. The analogy of a chin-up might be useful: a cycle of raising and lowering oneself on a chin-up bar results in no net work, but demands a large muscular work. Similarly, performing a slow upstroke while producing sufficient lift to support body weight requires negative work to be performed by the muscles; a wing-lifting force occurs at the same time as a wing-lifting motion. This work absorbed by the muscle must be replaced during the downstroke; the net work over the cycle may be small, but the muscle work high. To avoid this negative muscle work during upstroke while maintaining aerodynamic weight support, the wing cannot flap upwards too slowly. If the aerodynamic force is sufficient to lift the mass of the wings, but is not resisted by a moment at the shoulders (which would require energy dissipation), then the body of the bird (excluding the wings) should experience free-fall. This is supported by accelerometer measurements of geese and ibis during cruising flight (Fig. 5).

These muscle constraints provide an account for the benefit of the flap-gliding strategy. In birds with the capacity for large-amplitude, powerful flapping flight, a flap-gliding strategy during cruising flight may provide a physiological energetic advantage if:

1) flap amplitude is determined by a constraint to EMA modulation and the cost of low muscle strains in terms of excess muscle activation per work
2) flap speed is determined by a constraint to EMA modulation and the benefit of operating at a constrained range of muscle velocities
3) too-slow upstrokes while supporting body weight would result in negative work (and so – without some perfectly elastic mechanism – a greater positive work demand from the muscles during downstroke).

If the wing should be driven down deeply and quickly, and cannot be lifted too slowly, but near-continuous weight support is aerodynamically economical, periods of gliding are required. It should be noted that this account makes no prediction for the distribution of gliding periods: they could be achieved equally with a pause each upstroke or with a distinct separation between bouts of flapping and gliding.

## 6 Further discussion

### 6.1 Scaling of ‘gaits’

The developments presented here, while presented mainly as an account for bounding and flap-gliding flight strategies, also provide a broad account for the distribution of bird ‘gaits’. Early concepts of ‘gaits’ in bird flight focussed on the difference between birds not supporting body weight during upstroke – resulting in a gait that can be identified by the wake (Spedding et al., 1984) as a ‘vortex ring gait’ (Rayner, 1988) – and those that are – resulting in a ‘continuous vortex’ gait (Spedding, 1987, Rayner, 1988). More recent kinematic and aerodynamic observations (Hedrick et al., 2002, Spedding et al., 2003, Rosén et al., 2004, Tobalske et al., 2007) indicate that there is often no discernable discontinuity between these extremes, with the degree of weight support during upstroke effectively a continuous variable. However, consideration of the extremes remains useful. Why are vortex ring gaits, with aerodynamically inactive upstrokes, prevalent in small birds with high wingbeat frequencies? From Section 4.1, in order to ‘buy’ time to extend the duration of downstroke and reduce the muscle activation costs due to power, with the geometric constraints of providing thrust. Why do these issues apply less to larger birds in cruising flight, which are able to adopt something approaching a ‘continuous vortex’ gait with aerodynamic forces supporting weight throughout the upstroke? Both because they have sufficient time for their downstrokes to avoid excess muscle activation costs due to power; and because the geometric issues of orientating the net aerodynamic forces from the wings to provide thrust are reduced at larger sizes – parasite drag becomes relatively small compared with weight for bigger birds flying at similar absolute speeds. This leaves larger birds more able to adopt aerodynamically economical gaits.

The line of argument developed above contrasts with traditional accounts. Consider: large birds are *able* to adopt aerodynamically economical gaits because they flap with sufficient downstroke duration to avoid physiological costs of muscle activation due to power *versus* the conventional view that large birds are *constrained* to aerodynamically economical gaits due to the adverse scaling of aerodynamic force requirement (weight support) and production (wing area). Of course, both views may be valid, and may apply to greater or lesser extents depending on context. It is possible that the combination of these issues could provide further insight into functional implications of the remarkable diversity of form and function among flying birds.

### 6.2 When and why are intermittent strategies NOT adopted?

Can the issues proposed here account for cases of small flapping fliers *not* adopting bounding, and large birds *not* flap-gliding? Most insects and hummingbirds are considerably smaller than, or overlap in size with, bounding birds. This may be attributed to either of two assumptions made for bounding birds that do not apply – or apply to a much lesser extent – to insects and hummingbirds. The first is the degree of specialization of the wing profile and musculature for aerodynamic lift production only on the downstroke: the constraint to flap velocity *V*_flap_ of Fig. 3 does not apply if a forward-directed force vector can be achieved during the upstroke during forward flight. The second is the assumption of broadly scale-invariant muscle properties. Adaptations for very small-scale flapping flight certainly do include fundamentally different muscles, exemplified by the asynchronous insect flight muscles that allow extreme contraction frequencies in mosquitoes etc. One further issue that must be conceded is behavioural: for instance, kingfishers, shore-birds and hummingbirds may be of a scale and physiology that would benefit from bounding, but may find greater utility in specializing flight over flat surfaces, or hovering steadily at a food source.

The distribution of flap-gliding strategies among large birds may similarly have ecological or behavioural influences; for instance, many flap-glide specialists happen also to be capable soarers. However, the assumptions adopted here to account for flap-gliding do generally appear to tie in with the features of flap-gliding birds: flap-gliders appear to be those operating with a high ‘power margin’ or a high range of flapping flight speeds. This appears to apply to the contrast between large eagles and swans: eagles are capable of powered take-off from the ground while carrying prey loads; swans require a long running take-off. Similarly, the powerful burst-coast flight typical of phasianids (e.g. Tobalske and Dial, 2000) may be considered a form of flap-gliding exemplifying the advantages of operating the exceedingly powerful (Askew et al., 2001) muscles under favourable conditions, albeit only briefly or intermittently. Note that the account for flap-gliding does not have strong scaling predictions; there is no reason why small birds might not also benefit from adopting flap-gliding, particularly during slow flight. This may be consistent with observations of swallows and starlings, which adopt a speed-dependent range of intermittent flight strategies including flap-gliding.

### 6.3 Scaling of formation flight

There is increasing evidence (e.g. Portugal et al., 2014) that V-formation flight involves beneficial aerodynamic interaction between a following bird and the wake of a leader. V-formations appear to be limited to relatively large species. Presumably the absence of V-formation flight among small birds can, at least in part, be related to wake structures and, ultimately, to the flight styles determined by the physiological and geometric issues described here; it would appear very difficult to benefit from the wake produced by high-amplitude flapping and approximately ballistic periods during upstroke and/or bounding, especially by a bird following with a similar flight style.

### 6.4 Biomimetics

There is considerable military, commercial and recreational interest in aerial platforms that overlap in size and capability with birds. The conclusion of this paper is that intermittent flight strategies may arise in part due to the costs associated with muscle activation. It would therefore appear sensible to avoid biomimicry of either bounding or flap-gliding flight, even in such platforms that make use of flapping, because most actuators used in engineering do not have a similar activation cost.

### 6.5 Conclusion and predictions

Inclusion of a cost of muscle activations appears to provide a novel, general prediction of physiological benefits to high-amplitude flapping with little weight support during upstroke among small birds, and both bounding and flap-gliding flight strategies. Bounding is predicted to be associated especially with small birds, and relatively long bound durations are predicted at higher speeds and with higher parasite drag coefficients due to geometric issues of producing thrust. Flap-gliding at cruising speed is predicted in larger fliers also competent at flapping with high amplitude, fast wing strokes.
